# Pharmacotherapy post‐cardiac resynchronization therapy: Long walk home

**DOI:** 10.1002/ejhf.3796

**Published:** 2025-08-14

**Authors:** David Žižek, Marta Cvijić, Mitja Lainscak

**Affiliations:** ^1^ Cardiology Department University Medical Centre Ljubljana Ljubljana Slovenia; ^2^ Faculty of Medicine University of Ljubljana Ljubljana Slovenia; ^3^ Division of Cardiology General Hospital Murska Sobota Murska Sobota Slovenia

**This article refers to ‘Cardiac resynchronization therapy for enabling guideline‐directed medical therapy optimization in heart failure’ by D. Tomasoni *et al*., published in this issue on pages 1820–1833.**

Guidelines recommend individualized target or the maximally tolerated dose of heart failure (HF) pharmacotherapy.^1^ Despite challenges, strategies for titration of guideline‐directed medical therapy (GDMT) are well defined^2^, ^3^; however real‐world implementation of GDMT across HF phenotypes remains suboptimal.^4^ Multiple factors may contribute, including physiological parameters (e.g. blood pressure, heart rate, renal function), pharmacotherapy side effects, comorbidities, limited healthcare resources, as well as provider aversion and therapeutic inertia.^3^, ^4^ Cardiac resynchronization therapy (CRT), a non‐pharmacological HF modality, abolishes the risk of bradycardia, improves cardiac mechanical function and consequently the cardiac output, which translates to improved patient well‐being and prognosis. Effectively, this should prompt clinicians to fine‐tune the HF pharmacotherapy from maximally tolerated dose to target dose or to enable pharmacotherapy initiation. Unfortunately, the information whether and how this is implemented in daily practice is limited.

Registries contain wealth of information that can be utilized in numerous ways. The Swedish HF Registry is a good example how to advance the current knowledge and plan future research. For the issue of post‐CRT pharmacotherapy, the work by Tomasoni *et al*.^5^ is another example of pioneering work to evaluate current situation and guide activities to come. Using data from more than 6000 patients in the Swedish HF Registry and Swedish Pacemaker and Implantable Cardiac Defibrillator Registry, the authors nicely demonstrate that patients implanted with CRT were significantly more likely to have the HF pharmacotherapy optimized, particularly for beta‐blockers and diuretics (*Figure* *1*). Although the study did not specifically addressed improvement in cardiac function, these findings align with the concept that CRT can reverse electrical and structural dyssynchrony,^6^ thereby improving tolerability of medications that often worsen hypotension or renal function, while reducing the need for diuretics. CRT may also alter drug pharmacokinetics and dynamics by improving systemic perfusion.^3^, ^7^ This synergistic role of CRT as both a device therapy and a pharmacologic enabler is particularly relevant in light of the patient profiling framework of the Heart Failure Association.^3^

**Figure 1:**
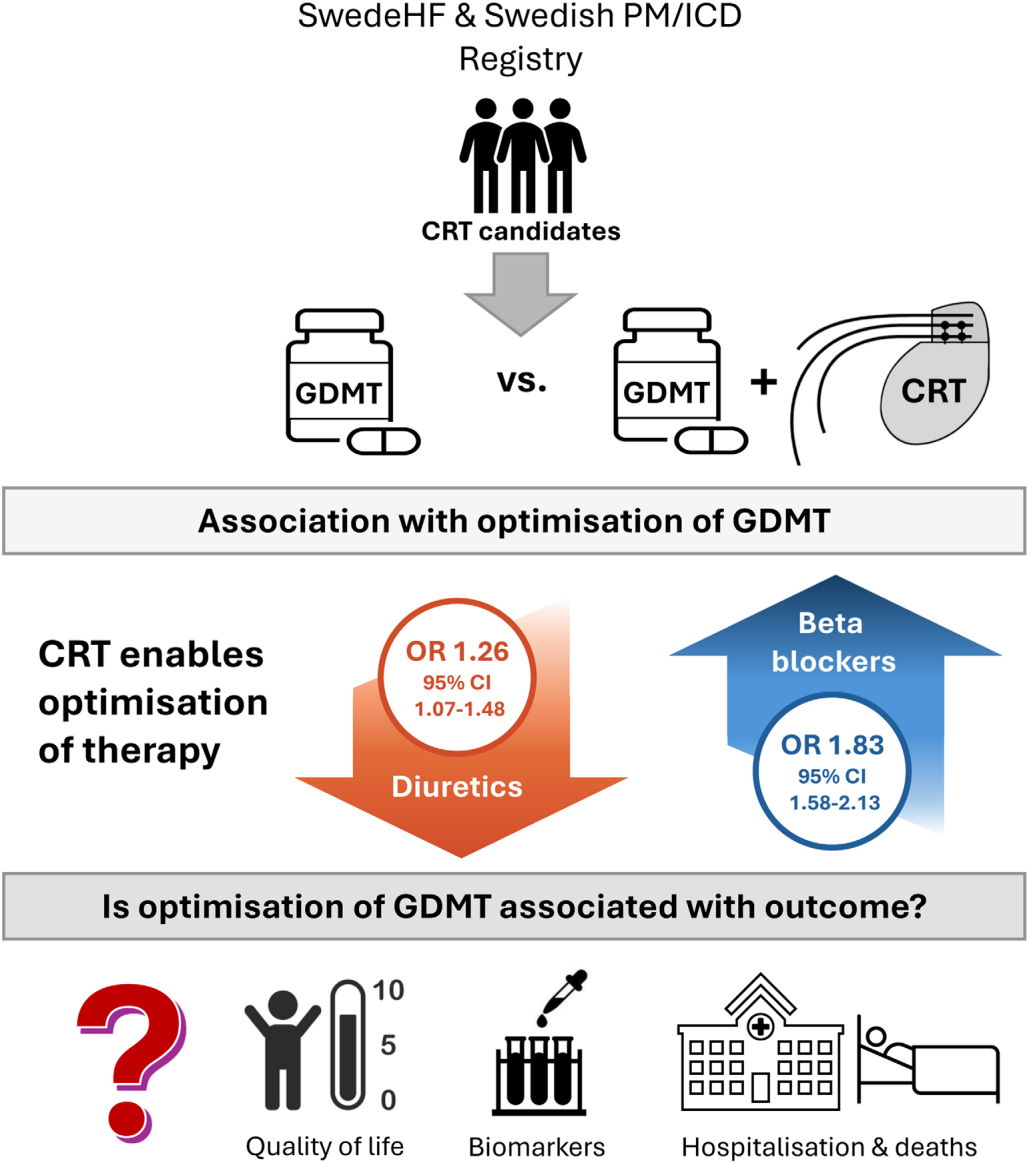
Cardiac resynchronization therapy as an enabler for guideline‐directed medical therapy (GDMT). CI, confidence interval; CRT, cardiac resynchronization therapy; HF, heart failure; ICD, implantable cardioverter‐defibrillator; OR, odds ratio; PM, pacemaker.

This study also highlights persistent therapeutic gaps that are evident in real‐world clinical practice. Although Tomasoni *et al*.^5^ showed that patients with CRT were almost twice as likely to have beta‐blockers up‐titrated when compared to patients without CRT, beta‐blocker up‐titration occurred in less than half of CRT recipients, and less than half received target doses. Herein, it would be interesting to understand the reason for failure to up‐titrate the beta‐blocker or not prescribe the target dose in CRT patients, especially since bradycardia was no longer a limitation. One issue with these registry data is establishing the reasons why the target dose was not achieved and identifying the rate of contraindications and intolerance to GDMT. The target dose of GDMT is an important goal in the management of HF patients and should be attempted for most patients.^2^ However, it is also crucial to determine the optimal dose for an individual HF patient when the target dose cannot be tolerated. Appropriate documentation of the highest‐tolerated dose would provide better insight into GDMT utilization rates and might better identify the patients who are eligible for further up‐titration.

Additionally, authors also reveal the issue of underutilization of CRT itself, although this is not explicitly stated.^5^ Despite class I indications, as many as two‐thirds of eligible patients were not referred for CRT implantation. This proportion is surprisingly high, particularly given that Sweden is in the highest quartile of CRT device implantations per million inhabitants in Europe.^8^ A joint position statement from the three European Society of Cardiology Associations has explicitly called for improved referral strategies and integration of CRT into standard HF care.^9^ This can be achieved by raising awareness of CRT among non‐specialists, improving geographic disparities in implant availability, and reducing systemic inertia in referral pathways. It is worth noting that this underuse of CRT represents a missed opportunity not just for mechanical benefit, but also for the broader goal of optimizing GDMT. Indeed, evidence from smaller studies suggests that a proactive strategy focusing on HF medication optimization, in addition to device programming and arrhythmia management, confers incremental benefits in terms of reverse remodelling and survival following CRT implantation.^10^, ^11^

We underline that the publication by Tomasoni *et al*.^5^ is important but leaves several aspects open; some of those could be within the registry reach and hopefully will be addressed in the future. Prior to CRT implantation, the target dose of GDMT was more likely prescribed in those receiving CRT. This opens the question of whether all patients had been adequately treated with GDMT and whether they were exposed to comparable active up‐titration during the follow‐up period. A previous study nicely demonstrated that an HF clinic staffed by specialized nurses and pharmacists could successfully increase the proportion of patients receiving therapeutic doses of GDMT within a relatively short follow‐up period.^12^ It would also be relevant to have more granular information whether CRT modality (with or without defibrillator), heart rhythm (sinus rhythm or atrial fibrillation), QRS duration and morphology were relevant in the context of HF pharmacotherapy. The message of this paper is focused on pharmacotherapy optimization but leaves the community without information whether this has translated into better quality of life, lower biochemical HF markers and improved prognosis in terms of hospitalization and mortality (*Figure* *1*). These aspects deserve to be addressed, either from the Swedish registries or others who have the capacity to deliver.

While awaiting future analyses, clinicians must make every effort to identify patients eligible for CRT and optimize management after implantation. This approach is both physiologically sound and clinically feasible. With the pioneering work of Tomasoni *et al*.^5^ in this field, we hope they continue to provide valuable data and inspire others to contribute as well. Additionally, it is important to see initiatives that support the implementation of the best available evidence into clinical practice rather than solely focusing on cutting‐edge science. A question arises regarding who should promote these actions. The scientific community, despite lacking a stable long‐term funding source, should not simply remain passive. Instead, through active collaboration with relevant funding bodies, we can create initiatives like JACARDI (Joint Action on Cardiovascular Diseases and Diabetes), which is the second‐largest European Union action featuring 142 pilot projects across 18 European Union countries.^13^ Such initiatives are crucial given the diversity of therapeutic approaches across Europe and could significantly transform HF management on a larger scale. The European Society of Cardiology and the Heart Failure Association have the potential to lead such efforts.

## Funding

D.Ž., M.C., and M.L. are funded by Slovenian Research and Innovation Agency (grant no.: P03‐456).

**Conflict of interest**: none declared.
